# Graphene oxide and starch gel as a hybrid binder for environmentally friendly high-performance supercapacitors

**DOI:** 10.1038/s42004-021-00604-0

**Published:** 2021-12-06

**Authors:** Mario Rapisarda, Frank Marken, Michele Meo

**Affiliations:** 1 Department of Mechanical Engineering, University of Bath, Bath, BA27AY UK; 2 Department of Chemistry, University of Bath, Bath, BA27AY UK

**Keywords:** Supercapacitors, Biopolymers, Electronic properties and devices

## Abstract

Alternative green binders processable in water are being investigated for the development of more efficient and sustainable supercapacitors. However, their electrochemical performances have fallen within or below the average of commercially available devices. Herein, an optimised gelled mixture of graphene oxide (GO) and starch, a biopolymer belonging to the family of polysaccharides, is proposed. The molecular interactions between the two components enhance electrodes structure and morphology, as well as their thermal stability. GO, thanks to its reduction that is initially triggered by reactions with starch and further progressed by thermal treatment, actively contributes to the charge storage process of the supercapacitors. The optimised electrodes can deliver a specific capacitance up to 173.8 F g^−1^ while providing good rate capabilities and long-term stability over 17,000 cycles. These are among the best electrochemical performances achieved by environmentally friendly supercapacitors using a biomaterial as a binder.

## Abstract (web summary)

Environmentally friendly binders for energy materials may improve sustainability, but can suffer from poor performance. Here a gel derived from graphene oxide and starch is used as a hybrid binder for supercapacitors, providing good rate performance and stability over 17,000 cycles.

## Introduction

The on-going growth in global energy demand and the current transition toward a greener more sustainable industry are driving the research for the development of more efficient and sustainable energy storage devices^1^. A viable solution is represented by electrochemical double-layer capacitors (EDLCs), a type of supercapacitors accumulating and delivering electrical energy by ultra-fast electrostatic charge storage and release in the electrical double layer. Their rapid charge/discharge, high power density, cycling life, energy efficiency, broad operating temperature range, and scalable design allow EDLCs use in either electronic and portable devices, as well as for transportation and stationary applications^2^. Although their well-known drawback is a limited energy density, extensively addressed via multiple approaches^3–6^, concerns over the environmental impact of EDLC electrodes manufacturing process are only recently arising. In particular, commonly used binders are fluoropolymers as poly(tetrafluoroethylene) and poly(vinylidene fluoride) dispersed in organic solvents as *N*-methyl-*2*-pyrrolidone and acetonitrile. Both binder and solvent represent a major cost^7^ and, especially, the main source of danger to the environment^8–10^. For this reason, alternative binders consisting of biopolymers processable in water (i.e., “green binders” that are intrinsically renewable and biodegradable) could ultimately lead to cheaper and more environmentally friendly devices^11,12^. In fact, they would allow for a reduction in the complexity of the coating process, as there would be no need for a solvent entrapment and recovery system during the drying, and a mitigation of risks of environmental pollution, thanks to the complete absence of toxic compounds.

Starch (St) is an inexpensive and readily available biopolymer belonging to the class of polysaccharides. It is composed of two types of molecules, the linear and helical amylose and the branched amylopectin, which distribution depends on the native biomass. Non-edible potatoes are commonly used and lead to ~20 wt% amylose and ~80 wt% amylopectin^13^. For the peculiar physicochemical structure, starch represents an ideal candidate as a green binder^12,14,15^. While amylose behaves as conventional synthetic polymers and allows hydrophobic binding^16^, amylopectin proved to be the key to overcome brittleness and shrinkage of coatings^15^. Although being naturally insoluble in water, starch becomes easily processable upon heating at its gelatinisation temperature (~60–80 °C) due to the swelling of its granules and the solubilisation of amylose^17^.

Varzi, Passerini and Ruschhaupt^15,18^ proved that starch, or its mixture with guar gum, could effectively be used as alternative green binder, superseding carboxymethyl cellulose (i.e., the state of the art aqueous and non-fluorinated binder alternative^18^) issue of low mass loadings due to brittleness and shrinkage upon drying. They reported a specific capacitance of ~50 F g^−1^ in an organic electrolyte within a potential window of 0–2.5 V. Their work inspired Jeżowski and Kowlczewski^14^ to develop a starch-based conductive glue for an optimised coating process that improved high power performances, reporting an energy density of ~20 Wh kg^−1^ at ~10 kW kg^−1^ within 0–2.5 V. It is worth noting, however, that the findings of the aforementioned studies mostly rely on qualitative evaluations, and that the reported electrochemical performance falls within or below the average of commercially available devices^19^.

Graphene oxide (GO) is a functionalised form of graphene having oxygen groups, such as hydroxyl, carbonyl, alkoxy and epoxy, which disrupts the conjugated network of the sp^2^ lattice of carbon atoms^20,21^. The latter is responsible for the remarkable electronic and thermal properties reported for pristine graphene^22,23^. GO is capable of forming stable suspensions in water and can be templated in various assemblies with low cost and in environmentally friendly processes^24^. This allows its use for various applications such as aerogels for acoustic absorption^25^ and water treatment^26^, free-standing papers for multifunctional polymeric composites^27^ and EMI shielding^28^, and carbonaceous composites for SC electrodes^29^. In particular, Choi et al. recently exploited the inclusion of 2 wt% reduced GO (rGO) as an active binder in biomass derived activated carbons (ACs), where the remarkable electrochemical performances were attributed to the development of electrically conductive networks formed by the rGO binder and the pseudocapacitance contribution due to residual oxygen functional groups^30^. GO was also used as the precursor to fabricate crumpled graphene papers by Zhao and co-workers, where the excellent rGO mechanical stability was the key to fabricate all-solid-state and stretchable SCs for un-conventional electronic devices (i.e., wearables and portable)^31^. Moreover, the ability of oxygen grups on GO sheets to interact with polymers have been exploited to improve their electrical and mechanical properties, as well as their thermal stability^32^. In particular, biocomposites of starch and GO have recently been investigated^33–35^.

In this study, a hybrid green binder was obtained from the gelation of an optimised mixture of starch and GO (GO-St-gel). Hydrogen bonds between amylose, amylopectin, and oxygen functionalities on GO sheets were formed during the water processing and an extended thermal stability was achieved, as revealed from a physicochemical characterisation. The proposed binder, after its mixing in water with ACs and carbon blacks (CBs), was capable to form homogeneous carbonaceous coatings (GO-StC) with a 3D morphology that enhanced the charge transfer process in the manufactured electrodes. GO actively contributed to the electrochemical performance (hence “hybrid”) due to its partial reduction that started during the gelation, and that was later optimised thanks to a further thermal treatment. The assembled symmetrical EDLCs provided a high specific capacitance of 173.8 F g^−1^, good rate capabilities, and a remarkable long-term stability with a capacitance retention of 93.1% after 17,000 charge/discharge cycles. Considering also that the electrodes were fabricated following an industrial-ready manufacturing process, the hybrid and green GO-St-gel binder presented in this work is an ideal candidate for the development of environmentally friendly and high-performance energy storage devices.

## Results and discussion

### Fabrication and characterisation of GO-StC electrodes

GO-StC electrodes were fabricated by a conventional method consisting of three main steps. The synthesis of a GO and starch gel that served as a green and hybrid binder, then the preparation of a carbonaceous slurry by adding an active material, AC, and a conductive additive, CB, and finally a controlled coating on a current collector, the rGO paper, immediately followed by a drying process. All slurries were prepared so that the mass ratio of the components is fixed as AC:CB:GO-St = 85:5:10 by using essentially the same method and are termed GO-StC-I, GO-StC-II, GO-StC-III, GO-StC-IV, GO-StC-V, depending on the initial composition of the GO-St-gel (GO amount corresponding to the 2.5, 3.3, 5, 6.7, 7.5 wt%, respectively). Reference samples, with the binder consisting of St only (StC) and of GO only (GO-C), were also manufactured by a similar method. Slurry composition of different GO-StC and reference electrodes are summarised in Supplementary Table 1. It is crucial to note that the physicochemical characterisation was focused on the GO-StC-III electrode (containing 5 wt% GO) that provided optimised electrochemical properties.

**Fig. 1:**
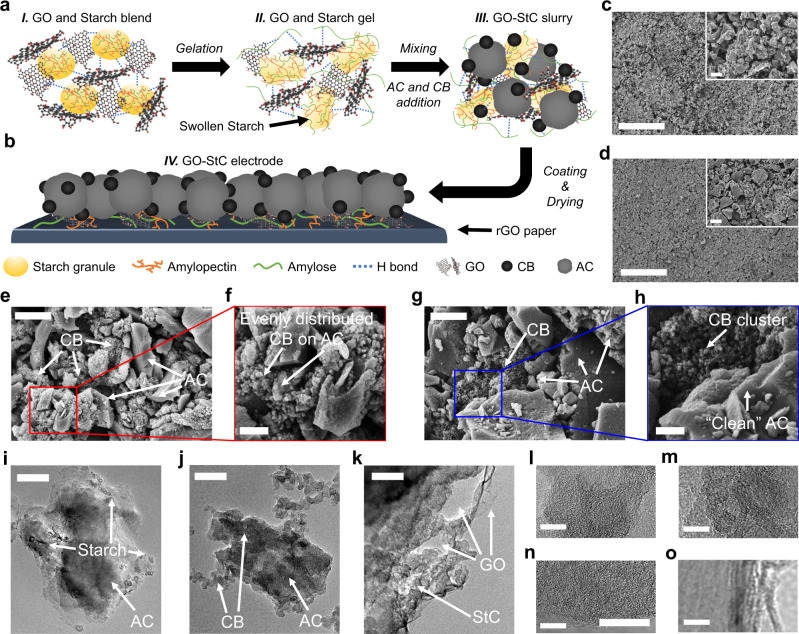
Schematic representation and electronic microscope imaging of GO-StC electrodes. **a** Schematic of GO-StC slurry preparation and of **b** GO-StC electrode. **c**–**h** SEM images showing: surface morphology of **c** GO-StC and **d** StC coatings (scale bar 100 µm) with higher magnification inset (scale bar 5 µm); AC and CB particles distribution in **e**, **f** GO-StC and **g**, **h** StC electrode materials (scale bars 2 µm and 500 nm). **i**–**o** TEM images of GO-StC electrode material showing: **i** starch particles attached to AC, **j** AC and CB particles distribution, **k** GO sheets at the base of the StC agglomerate (scale bars 200 nm), and **l** AC, **m** Starch, **n** CB and **o** GO magnified structures (scale bars 10 nm).

As shown in Fig. 1a, when mixed in water, GO sheets intercalates between starch granules thanks to the formation of hydrogen bonds between the molecules of the two components^33,36^. After heating the blend at a temperature of 70 °C, starch granules swell releasing amylose, mostly, and amylopectin molecules that can generate an amorphous network (i.e., gelation) and thus give the aqueous blend a viscoelastic behaviour^37^. On cooling, the disrupted chains usually tend to reassociate forming microcrystalline regions (i.e., retrogradation)^38^. However, due to the presence of GO intercalated sheets and their interaction through hydrogen bonds, the retrogradation can be inhibited^33^. Additionally, it was recently found that chemical reactions between starch molecules and oxygen functionalities attached to GO can lead to the partial restoration of the conjugated network of the sp^2^ lattice of carbon atoms and, consequently, the recovery of graphene electronic properties (i.e., reduction)^34,39,40^. The latter is believed to provide for a reduced electrical resistance and an improved electrochemical capacitance of fabricated electrodes^29,41,42^. Upon adding CB and AC particles, a homogeneous slurry is obtained, with the key role of linear amylose chains and GO sheets as dispersing agents^11,29,43,44^.

GO-StC electrodes are finally obtained with the subsequent coating and drying, as schematised in Fig. 1b. The resulting surface morphology can be observed from scanning electron microscopy (SEM) images (Fig. 1c and Supplementary Fig. 1a), where the effective role of branched amylopectin chains to build homogeneous coatings with limited shrinkage is proved^15^. Moreover, GO presence determines a more 3D structured morphology with respect of the reference StC coating (Fig. 1d and Supplementary Fig. 1b), that also shows some lumps due to a poorer dispersion of carbon particles. Higher magnification SEM images ultimately confirm the even distribution of CB particles among AC in GO-StC coatings (Fig. 1e, f), whether CB clusters near “clean” AC particles are found in StC (Fig. 1g, h). transmission electron microscopy (TEM) images show how Starch, AC and CB particles are distributed to form an StC agglomerate (Fig. 1i, j), with GO sheets being found at its base (Fig. 1k). Higher magnification TEM images of AC, Starch, CB and GO picture their distinctive structures (Fig. 1l–o).

**Fig. 2:**
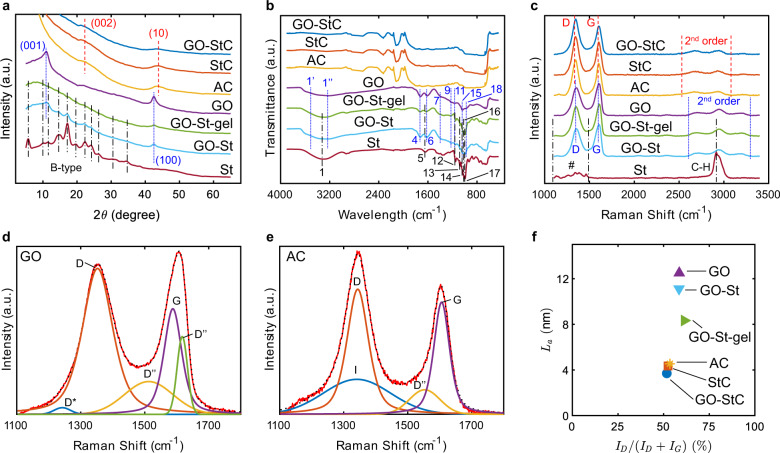
Physicochemical characterisation. **a** XRD patterns, **b** FT-IR, and **c** RS spectra of GO-StC electrode material and GO-St-gel binder compared with reference materials. Red dashed, blue dotted and black dashed and dotted lines refer to AC, GO and St related features, respectively. “#” stands for “Amylose and Amylopectin”. **d** GO and **e** AC deconvolution results. Red solid and black dotted lines indicate experimental measurements and fitting results, respectively. **f** Variation of *L*_a_ with *I*_D_/(*I*_D_ + *I*_G_) ratio.

In X-ray diffraction (XRD) patterns (Fig. 2a), GO shows the characteristic (001) peak associated to the carbon crystalline phase and the (100) peak related to the lateral dimension of basal planes at 10.80° and 42.57°, respectively^45,46^. St has instead the typical features of the B-type crystalline form, commonly found in potato starches with high contents of amylose (~20%)^47^. When GO is mixed with St (GO-St) the (001) peak disappears, indicating the absence of any ordered structure, usually due to the re-stacking of GO sheets driven from Van der Waals interations, and thus suggesting their intercalation between starch granules^48^. Moreover, after the gelation process the main features of starch are absent. This confirms the formation of an amorphous phase due to the release of amylose from swollen granules^49^, and with GO inhibiting the retrogradation into a crystalline matrix^33^.

XRD patterns of GO-StC electrode active material show the characteristic (002) and (10) peaks at 21.89° and 43.73°, respectively. The first derives from the interlayer spacing of graphene sheets (*d*) and is downshifted with respect of both StC (22.24°) and pure AC (22.13°), indicating, according to Bragg’s law, a slightly more expanded carbon structure (4.06 Å against 3.99 and 4.01 Å, respectively). The (10) peak derives instead from the merging of (100) and (101) lattice peaks typically shown in ordered graphite, as a consequence of turbostratic arrangements of graphene layers^50^. From Scherrer’s formula^51^, (10) and (100) peaks data related to AC and GO, respectively, are processed to estimate crystallites lateral size (*L*_a_).The reduction observed from GO (128 Å) to GO-St (109.6 Å) and GO-St-gel (86.9 Å) can be attributed to the rupture of GO sheets during the processing. On the other hand, GO-StC electrode material shows a smaller value (37.6 Å) with respect of both reference StC (43 Å) and starting AC (44.6 Å), explainable with a finer particle distribution due to GO sheets interactions. A summary of main peaks position and calculated structural parameters can be found in Supplementary Table 2.

Comparing main starch features in Fourier-Transform Infrared Spectroscopies (FT-IR) of GO-St-gel to GO-St and St (Fig. 2b), with particular regards in the disappearance of the 1642 cm^−1^ band (5, associated to water absorbed in the amorphous region), the disappearance of the 1042 cm^−1^ band (14, typical of crystalline phase), and the increase of the intensity ratio between the 1016 and 995 cm^−1^ bands (16 and 17, respectively due to C–OH bending and C–O–C skeletal vibrations)^52^, together with the shift of GO band at 1615 cm^−1^ (6, due to stretching and bending of OH groups in absorbed H_2_O molecules)^53^, ultimately provides for a successful gelation^49,54^. Moreover, a shift of OH related features for starch at 3310 cm^−1^ (1) and for GO at 1373, 1219 and 1165 cm^−1^ (7, 9 and 11, respectively) confirms the formation of hydrogen bonds between OH groups of both amylose and amylopectin and the oxygen functionalities on GO sheets^33,36^. Finally, from FT-IR spectra of GO-St before and after the gelation, the decrease of GO oxygen functional groups between 3586 and 3216 cm^−1^ (1′ and 1″, associated to OH stretching), and at 1720, 1038 and 975 cm^−1^ (4, 15 and 18, respectively attributed to C=O stretching of Carbonyl groups, C–O stretching of Alkoxy groups and C–O–C stretching of Epoxy groups)^55^ can be interpreted as a partial reduction of GO by its reaction with starch molecules^39^. A summary of all the features of the discussed materials can be found in Supplementary Table 3. Both GO-StC and StC shows no significant variation with respect of pure AC (Fig. 2b).

The absence of starch features (amylose and amylopectin vibrational modes between 1100 and 1500 cm^−1^, and C–H vibrations at 2915 cm^−1^)^52^ in Raman Spectroscopies (RS) before and after GO-St blend gelation (Fig. 2c) is mainly due to the stronger excitation of carbon features of intercalated GO sheet, but can also be attributed to structural changes caused by the rearrangement of hydrogen bonds between water and starch molecules^56^. All carbonaceous samples show the typical D (~1348 cm^−1^) and G (~1607 cm^−1^) bands associated to *A*_1g_ breathing mode due to structural disorder and *E*_2g_ vibrating mode of crystalline Graphite^57^ as well as 2nd order scatterings (~2550−3300 cm^−1^) caused by turbostratic arrangements of graphene layers^58^, however a distinction has to be made in their interpretation due to different material nature. Focusing on D and G bands with the aim to calculate the *I*_D_/(*I*_D_ + *I*_G_) ratio expressing carbons’ structural disorder^46^, both GO-St-gel and GO-St have been deconvoluted considering three additional interbands accounting for disorder (D*, D″ and D′, associated to sp^2^–sp^3^ bonds at the edges of networks, interstitial defects in amorphous lattices and phonon mode due to crystal defects, respectively) following Claramut et al. method for GO^59^ (Fig. 2d). The resulting *I*_D_/(*I*_D_ + *I*_G_) value for GO-St-gel is 61.33%, increased with respect of both GO-St and GO (58.43% and 58.56%, respectively) and thus corroborates the partial GO reduction previously deduced from FT-IR results^40^. GO-StC and StC have instead been deconvoluted considering only two additional interbands (I, associated to impurity ions, and D″ again) following Cuesta et al. method for AC^60^ (Fig. 2e). The smaller *I*_D_/(*I*_D_ + *I*_G_) value for GO-StC (51.72%) compared to both StC and AC (52.43% and 53.42%, respectively) can be ascribed to an improved disaggregation and stabilisation of AC and CB particles thanks to their interactions with GO sheets^29,43^, as previously suggested from XRD results. Figure 2f pictures the described trend for *I*_D_/(*I*_D_ + *I*_G_) and its relationship with *L*_a_. A summary of deconvolution parameters and resulting *I*_D_/(*I*_D_ + *I*_G_) ratios for the carbonaceous materials can be found in Supplementary Table 4.

### Thermal behaviour of GO-StC coatings

**Fig. 3:**
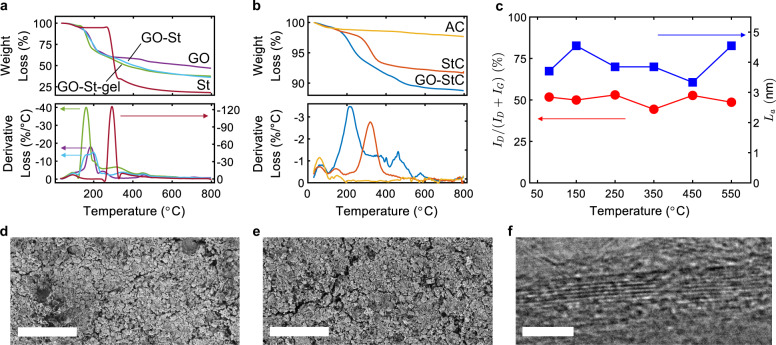
Thermal behaviour of GO-StC coatings. TGA and dTGA of **a** GO-St-gel, GO-St, GO, St and **b** GO-StC, StC, AC. **c** Variation of *I*_D_*/*(*I*_D_ + *I*_G_) ratio and *L*_a_ with the temperature. SEM images showing development of cracks after thermal treatment of GO-StC electrodes at **d** 350 and **e** 550 °C (scale bars 100 µm). **f** TEM image showing reduced GO after thermal treatment at 350 °C (scale bar 2 nm).

Thermogravimetric analysis (TGA) and derivative (dTGA) curves for the GO-St binder before and after the gelation and of GO-StC coatings with the relative reference materials are presented in Fig. 3a, b. Thermal reduction of GO in the gelled binder starts at a lower temperature (122 °C) with respect of pure GO (150 °C) and with a quicker kinetic with respect of GO-St. This behaviour is also followed by GO-StC (starting at 132 °C) and is attributable to chemical reactions between starch and GO molecules previously described. Starch, and thus binder, degradation starting is shifted to higher temperature from pure St (255 °C) to its blend with GO (286 °C), but it is anticipated after gelation (start at 250 and 240 °C for GO-St-gel and StC, respectively). The starting point of the degradation is not clear in GO-StC, although it can be estimated being in a range between 250 and 300 °C with a kinetic that is nevertheless slower with respect of the electrode material fabricated without GO (StC). These results provide for a reciprocal beneficial effect of the interactions between GO and Starch, with a boost of GO reduction and an improvement of the thermal stability of the binder.

The thermal behaviour of the GO-StC coating was further analysed with XRD, FT-IR and RS (Supplementary Fig. 2a–c), after their thermal treatment at a temperature ranging from 150 to 550 °C for a total duration of 1 h under inert atmosphere. While FT-IR did not provide additional information, mainly because the material is mostly composed of inert carbons with strong absorbance bands, XRD and RS data were processed to evaluate the variation of *I*_D_/(*I*_D_ + *I*_G_) and *L*_a_ with the thermal treatment temperature. The results are presented in Fig. 3c and show that the disorder ratio, after an initial decrease (from 51.72 to 49.94% at 80 and 150 °C, respectively), reaches a maximum at 250 °C (53.02%) and then a minimum at 350 °C (44.37%), before increasing again for higher temperatures. *L*_a_ shows an inverse trend with respect of *I*_D_/(*I*_D_ + *I*_G_) at all temperatures but 350 °C and presents a minimum at 450 °C (32 Å). Such behaviour can be attributed to the subsequent processes of GO reduction and Starch degradation, which are superimposed in the range of 250−300 °C. A summary of main XRD peaks position and calculated structural parameters, and of RS deconvolution parameters and resulting *I*_D_/(*I*_D_ + *I*_G_) ratios for the thermally treated materials can be found in Supplementary Tables 6–7. Changes in coatings morphology with temperature were also observed with SEM imaging, with development of small of cracks at 350 °C (Fig. 3d) and their enlargement at 550 °C (Fig. 3e). Finally, TEM imaging shows the recovery of the graphitic structure of reduced GO after the treatment at 350 °C. As a result of the evaluation of the thermal behaviour of the GO-StC electrodes, the temperature of 350 °C appear to be the highest possible without affecting the structural integrity of the coatings. This is allowed by the residual oxygen functionalities in rGO after its partial reduction which preserve the bonding sites between rGO and Starch molecules, as indirectly confirmed by the evaluation of the resulting electrochemical properties discussed in the following paragraphs.

### Electrochemical performance of GO-StC electrodes

**Fig. 4:**
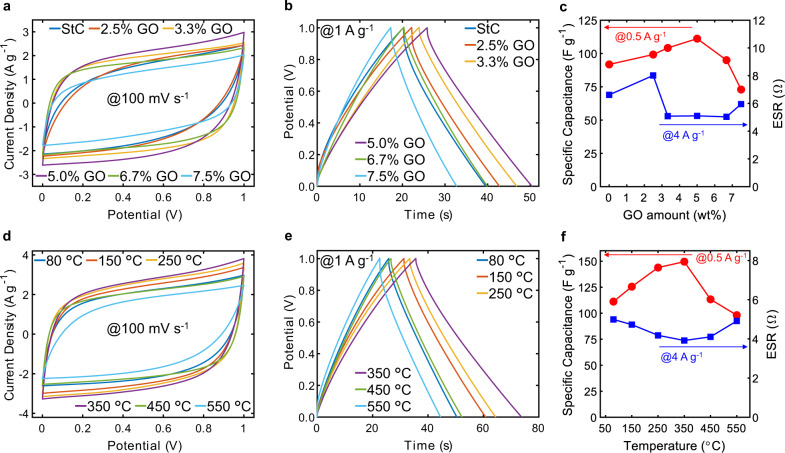
Electrochemical characterisation of supercapacitors. **a** CV curves at a scan rate of 100 mV s^−1^ and **b** GCD curves at a current density of 1 A g^−1^ for GO-StC electrodes with varying GO amounts. **c** Variation of specific capacitance and equivalent series resistance (ESR) with GO amount. **d** CV curves at a scan rate of 100 mV s^−1^ and **e** GCD curves at a current density of 1 A g^−1^ for GO-StC electrodes with varying thermal treatment temperatures. **f** Variation of specific capacitance and ESR with the temperature.

To understand the role of GO amount on electrochemical performance of GO-StC electrodes, all-solid-state supercapacitors were assembled as symmetrical cells using poly(vinyl alcohol) (PVA)/H_3_PO_4_ as electrolyte gel, which also behaves as separator, and the rGO paper as current collector. Cyclic voltammetry scans (CVs) at 100 mV s^−1^ for GO-StC electrodes with different GO contents ranging from 0% (StC) to 7.5% are shown in Fig. 4a. Electrodes with GO content of 10% (GO-C) have been excluded as the coatings did not possess acceptable structural robustness. Quasi-rectangular CVs can be observed from the 3.3%, as well as a progressive widening of subtended area up to the 5%. This is directly linked to an improved electrochemical capacitance and a reduced electrical resistance of the electrodes. The beneficial effect of GO on electrochemical performance of assembled supercapacitors is confirmed by the triangular and symmetric galvanostatic charge-discharge cycles (GCDs) at a current density of 1 A g^−1^ presented in Fig. 4b. The specific capacitance, calculated from GCDs discharge profile at 0.5 A g^−1^ (Supplementary Fig. 3), increased from 91.8 F g^−1^ of StC (without GO) to 111.2 F g^−1^ of the cell with 5% GO, and decreased for higher GO inclusions (Fig. 4c). On the other hand, the equivalent series resistance (ESR), calculated from the initial potential drop of GCDs discharge profile at 4 A g^−1^ (Supplementary Fig. 3), initially increased from 6.62 Ω of StC to 8.02 Ω for 2% GO, and then dropped to ~5.07 Ω for GO amount between 3.3 and 6.67% (Fig. 4c). The improved performance with the increase in GO content up to 5%, where it is present in the GO-St-gel binder in equal amount with starch, can be attributed to an indirect contribution of the optimised binder. Particularly, it promotes a transition from a planar and not perfectly homogeneous coating with poorly distributed CB clusters to a 3D structured morphology with evenly distributed CB particles among ACs, as previously discussed. Moreover, the enhanced charge storage capability upon GO inclusion could also derive from a direct contribution of fast red-ox reactions taking place near GO sheets due to the presence of oxygen functional groups^61^. The decrease in specific capacitance and increase in ESR occurred when GO amount was increased to 6.7 and 7.5%, respectively. As a consequence of the latter, St amount was reduced to only the 3.3 and 2.5%, respectively, of the electrode material leading to an insufficient presence of amylose and amylopectin molecules (i.e., the crucial components providing binding capability to the GO-St gel). Subsequently, the observed detrimental effect on both specific capacitace and ESR is likely due to a lack in both cohesion between carbon particles of the coating and in its adhesion on the current collector.

The electrochemical performance of optimised GO-StC electrodes with 5% GO inclusion were further investigated at varying thermal treatment temperatures from 80 °C (only-dried after coating) to 550 °C. CVs at 100 mV s^−1^ and GCDs at 1 A g^−1^ (Fig. 4d, e) describe an improved electrochemical behaviour up to 350 °C, and a clear drop in performance at higher temperatures. In particular, Fig. 4f shows the specific capacitance at 0.5 A g^−1^ reaching a maximum of 149.5 F g^−1^ at 350 °C before sharply decreasing to 113 and 98.12 F g^−1^ at 450 and 550 °C, respectively. The ESR at 4 A g^−1^ also reaches its optimum at 350 °C, with a value of 3.92 Ω (Supplementary Fig. 4). Performance improvement with thermal treatment, at temperature up to 350 °C, can be directly linked to the beneficial effects of GO reduction^42^. Upon heating, more oxygen functional groups and intercalated water molecules are released as CO_2_, CO and vapour, complementing the recovery of sp^2^ carbons lattice and, consequently, of electronic properties of graphene started during the gelation of the binder^40^. This directly accounts for the optimisation of both specific capacitance, due to the enhanced charge transfer process,^42,62^ and ESR, thanks to the improved electrical conductivity^63^. Conversely, the decrease of electrochemical properties after a thermal treatment at higher temperatures, namely 450 and 550 °C, can be ascribed to multiple factors affecting charge transfer. The first is undoubtedly starch degradation, which starting temperature was estimated at ~250−300 °C from TGA results and with clear evidence of coating structure cracking at 550 °C from SEM imaging, as previously described. Additionally, the exposure at higher temperatures leads to a more extensive reduction of GO and thus to a removal of all the residual oxygen functionalities that positively contributes to charge storage capabilities^61,64^. This explains the sharp drop in specific capacitance, while the less severe impact on ESR is likely due to a balance between the negative effect caused by the loss of cohesion and adhesion due to starch degradation, and the increased electrical conductivity thanks to GO reduction.

### High electrochemical performance of thermally treated GO-StC electrodes

**Fig. 5:**
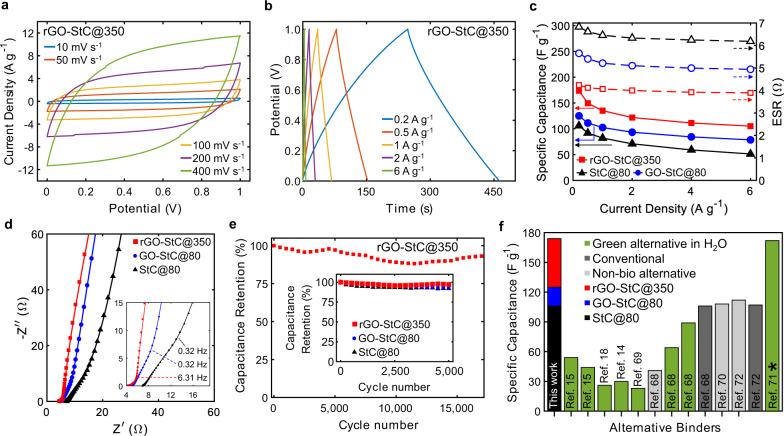
Electrochemical characterisation of optimised GO-StC electrodes. **a** CVs at scan rates of 10 to 400 mV s^−1^ and **b** GCDs at current densities of 0.2 to 6 A g^−1^ for GO-StC electrodes with 5% GO after thermal treatment at 350 °C (rGO-StC@350). **c** Variation of specific capacitance and ESR with current density and **d** Nyquist plots of rGO-StC@350 with dried-only GO-StC and StC electrodes (GO-StC@80 and StC@80) included for comparison. High-frequency region magnified in the inset. Markers represents the experimental points, while solid lines the modelled behaviour with the simplified equivalent circuit (Supplementary Fig. 6). **e** Capacitance retention during the cycling at 4 A g^−1^ of rGO-StC@350 °C up to 17,000 cycles. Inset showing the comparison with GO-StC@80 and StC@80 up to 5000 cycles. **f** Comparative bar chart expressing the high performance of the GO-St-gel (stacked bar coloured in black, blue and red when referring to StC@80, GO-StC@80 and rGO-StC@350, respectively) among all alternative green binders processable in water (coloured in green) and conventional binders of commercially available devices (coloured in dark grey). Some non-biomaterial based greener alternatives have also been included as reference (coloured in light grey). Water is used as the only solvent unless differently specified between brackets (*water is mixed with acetic acid).

The symmetrical cell assembled with GO-StC electrodes containing 5% GO and thermally treated at 350 °C (rGO-StC@350) resulted as the best performing supercapacitor. It was further evaluated through a full electrochemical characterisation, while dried-only GO-StC and StC electrodes (GO-StC@80 and StC@80, respectively) were considered for comparison. The rGO-StC@350 cell exhibits desirable charge storage capabilities and rate performance, as shown in quasi-rectangular CVs from 10 to 400 mV s^−1^ and symmetric triangular GCDs from 0.2 to 6 A g^−1^ (Fig. 5a, b). It also provides a high specific capacitance of 173.8 F g^−1^ at 0.2 A g^−1^ with a capacitance retention of 60.5% (105.2 F g^−1^) at the high scan rate of 6 A g^−1^ (Fig. 5c), where the minimum recorded ESR value of 3.89 Ω is found. GO-StC@80 and StC@80 cells both provides lower charge storage capabilities over all the tested currents, having a specific capacitance of 125 and 105.9 F g^−1^ at 0.2 A g^−1^ and of 78.5 and 51.2 F g^−1^ at 6 A g^−1^ (with ESRs of 4.94 and 6.18 Ω), respectively. It is also worth noting the increased rate performance from StC@80 to GO-StC@80, with capacitance retentions of 48.3 and 62.8%, respectively. According to Eqs. (6) and (7), the increase of specific capacitance and the reduction of ESR upon GO inclusion and its subsequent reduction at 350 °C determine the increase of the maximum gravimetric energy and power densities from 14.71 Wh kg^−1^ and 7.75 kW kg^−1^ (StC@80) to 24.14 Wh kg^−1^ and 14.48 kW kg^−1^ (rGO-StC@350) (values are summarised in the Ragone plot in Supplementary Fig. 5). Further details of the coatings and the resulting volumetric capacitance are summarised in Supplementary Table 8.

Electrochemical impedance spectroscopy (EIS) measurements were performed on selected electrodes at open circuit potential. The impedance behaviour of the symmetric cells (schematised in Supplementary Fig. 6a) was successfully modelled with the simplified equivalent circuit proposed in Supplementary Fig. 6b (fitting parameters are summarised in Supplementary Table 9). The resulting Nyquist plots (Fig. 5d) show the development of a depressed semi-circle in the high frequency region in both GO-StC@80 and rGO-StC@350, which is attributed to charge building up at the interface of the current collector with the coating. The straight line with slope of ~45° at middle frequencies can instead be related to charge transport within the porous electrodes (i.e., transmission line behaviour)^65^, with a much quicker transition toward the capacitive behaviour (almost vertical straight line at low frequencies) for rGO-StC@350 (occurring at 6.31 Hz, compared to 0.32 Hz of both StC@80 and GO-StC@80). As shown in Bode and complex imedance plots (Supplementary Fig. 6c–f), the middle frequency transition is consistent with the improved capacitor time response assessed from the frequencies leading to a phase angle of −45° in Bode plots and from relaxation frequencies corresponding to local maxima of the imaginary part of capacitance (frequencies value are reported in Supplementary Table 9). For all the tested symmetric cells, the low frequency values of the real part of capacitance is in agreement with results from GCDs. From fitted values of the simplified equivalent circuit elements, it is worth noting the decreasing trend of the series resistance (*R*_s_) from StC@80 (6.83 Ω) to GO-StC@80 (4.43 Ω) and rGO-StC@350 (4.12 Ω), as well as the increasing coefficient related to the phase and the parameter accounting for the capacitance of the constant phase element representing the double layer capaticance (*C*_dl−P_ and *C*_dl−T_, respectively). In particular, a phase value of 1 corresponding to an ideal capacitor behaviour was obtained for rGO-StC@350. EIS analysis thus further confirms the enhanced charge transfer dynamic upon inclusion and subsequent reduction of GO, and consequently proves a direct contribution of the binder to the electrochemical performances.

The rGO-StC@350 supercapacitor also possesses a remarkable cycling stability, with a capacitance retention of 93.1% after 17,000 GCDs at 4 Ag^−1^ (Fig. 5e). Its cycling performance after 5,000 cycles (97.1%) outperformed both GO-StC@80 (92.5%) and StC@80 (92%). Such behaviour confirms the structural integrity of the coatings is not affected by the heating at the designed temperature with reduced GO sheets capable of maintaining a fast and efficient charge storage process upon long-term cycling. Moreover, it is possible to speculate that the fluctuation observed in the capacitance retention could be due to the ability of graphene sheets and residual oxygen functionalities to generate optimised ion diffusion paths during the charge and discharge phases when electrolyte ions intercalates through the pores^66,67^.

All latter results confirm previous assumptions of the beneficial effects on electrochemical performance in GO-StC electrodes with an optimised inclusion of 5% GO into the gelled GO-St binder, which can further be enhanced by an optimised thermal treatment at 350 °C. Finally, the bar chart in Fig. 5f shows that the specific capacitance of the optimised rGO-StC@350 cell is the highest value reported for supercapacitors assembled with alternative green binders processable in water, also outperforming conventional binders used for commercially available devices and other non-biomaterial based greener alternatives (Supplementary Table 10)^14,15,18,68–72^.

## Conclusion

This work demonstrates that a hybrid and green binder can be obtained from the gelation of an optimised mixture of a polysaccharide biomaterial, starch, and GO sheets. Supercapacitors electrodes were then manufactured by a conventional and industrial-ready manufacturing process having water as the only ecological solvent and without any hazardous substances being ever involved, including in the polymer electrolyte gel. A further optimisation of the binder was achieved after a thermal treatment of the electrodes, where the reduction of GO sheets fully unlocked their capabilities to actively contribute to electrochemical performances. The physicochemical characterisation revealed the interactions between starch and GO and their positive effects on coatings structure and morphology, as well as thermal treatment consequences at varying temperatures. An extended thermal stability of the GO-St-gel binder was also proved. Nevertheless, the electrochemical characterisation of symmetrical and all-solid-state supercapacitor cells assembled with GO-StC electrodes demonstrated the ability of the studied binder to provide satisfactory cohesion between AC and CB particles and their adhesion on the rGO paper current collector, due to the crucial role of amylose and amylopectin molecules. Furthermore, the beneficial effects of the optimised amount of 5% GO, and of its reduction after the thermal treatment at the optimised temperature of 350 °C, on a fast and efficient charge storage process were clearly evidenced. The rGO-StC@350 cell presented in this manuscript provided a high specific capacitance of 173.8 F g^−1^ at 0.2 A g^−1^ (that is the highest ever reported when alternative green binders processable in water were used), good rate capabilities up to 6 A g^−1^ and a remarkable long-term stability with a capacitance retention of 93.1% after 17,000 charge/discharge cycles at 4 A g^−1^. For this reason, the adoption of the GO-St-gel as a hybrid and green binder is envisaged for the manufacturing of environmentally friendly and high-performance supercapacitors as a potential solution for the continuous growth of energy consumption and global pollution.

## Methods

### Materials

Graphite oxide (GtO) powder (SE2430) was purchased from Xiamen TOB New Energy. AC powder (HCE 202) was acquired from Haycarb PLC. Starch, PVA (98–99% hydrolysed, medium molecular weight) and H_3_PO_4_ (ACS reagent, 85 wt% in H_2_O) were purchased from Sigma Aldrich. CB powder (Black Pearls 2000) was supplied by Cabot Corporation. Deionized water (MilliQ) was used throughout all the experiments. All materials were used without further purification.

### Fabrication of GO-StC electrodes

#### GO-St gel synthesis

A GO aqueous dispersion (8 mg mL^−1^) was first obtained as follows: GtO powder (500 mg) and 50 mL of water were mixed thoroughly and probe ultrasonicated (Dr. Hielscher GmbH UP100H, with an amplitude of 80% and continuous pulsing) for 40 min, vigorous magnetic stirring and an ice bath ensured a homogeneous process with controlled temperature. Then, St granules (500 mg) were added into the GO dispersion and mixed thoroughly with bath ultrasonication for 10 min. Finally, the gelation was promoted by heating at 80 °C for 30 min with mild magnetic stirring. After cooling down, the gel was sealed in a vial and preserved at ambient conditions for further use. Samples of GO dispersion, GO-St blends before and after gelation and of the final gel were drop casted onto quartz glass slides and dried at ambient conditions for their physicochemical characterisation.

#### GO-StC slurry preparation

Well-ground CB powder (500 mg) and 50 mL of GO-St hydrogel were mixed thoroughly with overhead stirring (Heidolph RZR 50) for 1 h. Then, well-ground AC powder (8,500 mg) was slowly added into the black slurry and overhead stirred for 12 h. Finally, the viscous slurry was sealed in a vial and preserved at ambient conditions for further use. The reported amount of materials refers to the optimized GO-StC-III slurry. Different amount of St, CB and AC were used to obtain GO-StC slurries with varying GO contents from 2.5 to 7.5 wt% with respect of the total mass of solids. For comparison, a GO-C and a StC slurry were prepared by sequentially adding CB and AC to the GO dispersion (i.e., without starch and with GO accounting for the 10 wt%) and to a St-only hydrogel (i.e., without GO and with starch accounting for the 10 wt%), respectively.

#### rGO paper preparation

The rGO paper was prepared as previously reported^27^. Briefly, a GO aqueous dispersion was cast on a polymeric film and dried at ambient conditions. Then, the obtained GO film was peeled off and annealed at 1,300 °C for 3 h under inert atmosphere. Finally, the obtained rGO film was calendered to obtain a freestanding paper with a thickness of 40 µm and a density of 1.18 g cm^−3^.

#### GO-StC electrode fabrication

The GO-StC slurry was coated on the rGO paper using an adjustable film applicator with a set thickness of 200 µm and vacuum dried at 80 °C for 1 h. Thermally treated electrodes were obtained with further heating at varying temperatures from 150 to 550 °C for 1 h under Argon atmosphere. Finally, the resulting electrodes, having a mass loading of ~2.8 mg cm^−2^ and a thickness of ~114 µm, were cut to squares of 2 cm side and preserved in a dry atmosphere for further use. Powder samples of the coatings were scraped from the substrate and used for their physicochemical characterisation.

### Physicochemical characterisation

#### Physicochemical testing

The surface morphology and microstructure of GO-StC and StC electrodes was observed using SEM (JEOL JSM-7900F) and TEM (JEOL JEM-2100Plus). The crystal phase composition was analysed by transmission powder XRD (STOE STADI P) using a CuKα generator. The chemical structure was investigated with FT-IR (Perkin-Elmer Frontier FTIR Spectrometer) using a MCT detector and with RS (Renishaw inVia Raman microscope) using a 532 nm laser source (IK Series He-Cd). The thermal stability was evaluated by TGA (Setaram SETSYS Evolution 16 TGA-DTA/DSC).

#### Physical and chemical parameters calculations

XRD data were used to estimate *d* from the (100) or (200) bands and *L*_a_ from the (10) or (100) bands, using Bragg’s law^73^ and Scherrer’s formula^51^, respectively:

1 $$d = \frac{\lambda }{{2\,\sin \theta }},$$

2 $$L_{{{{{\mathrm{a}}}}}} = \frac{{K\,\lambda }}{{B\,\cos \theta }},$$

where *λ* is the radiation wavelength, *θ* is the corresponding scattering angle of the lattice (depending on carbon material’s nature), *K* is a shape factor (equal to 1.84 for carbon materials^74^), and *B* is the line broadening at half-maximum intensity of the peak. Raman spectra were processed with multiple interbands deconvolution, as discussed in the manuscript, to obtain the *I*_D_/(*I*_D_ + *I*_G_) index expressing carbons’ structural disorder^46^.

### Electrochemical characterisation

#### Supercapacitor assembly

A typical symmetrical cell was assembled with two identical GO-StC electrodes with the rGO paper serving as the current collector. A PVA/H_3_PO_4_ gel was employed as both the separator and the electrolyte. First, a PVA solution (10 wt%) was prepared dissolving PVA granules (5 g) in water (50 mL) with continuous stirring and heating up to 90 °C for 4 h. Then, H_3_PO_4_ (7.5 g) was added to the clear solution and stirred for 12 h to obtain the PVA/H_3_PO_4_ electrolyte gel. Electrical connections were provided by copper tape attached to the end of each electrode using silver paste and protected with Kapton tape masking, so that an active area of 1 cm^2^ was obtained. Then, a drop of the electrolyte gel was casted on the electrodes and dried for 4 h. Finally, two identical electrodes were sandwiched with a drop of the electrolyte gel and dried overnight.

#### Electrochemical testing

Electrochemical performances were measured using an electrochemical workstation (Solartron ModuLab XM MTS) in two-electrode configuration. CVs, with scanning rates ranging from 10 to 400 mV s^−1^_,_ and GCDs, at current densities ranging from 0.2 to 6 A g^−1^_,_ were performed with a potential ranging from 0 to 1 V. Cycling stability tests were conducted by GCDs at a current density of 4 A g^−1^. EIS measurements were acquired over a frequency range of 10 mHz to 100 kHz with an amplitude of 10 mV at the open circuit potential.

#### Electrochemical performance calculations

Electrochemical performance calculations were all performed from discharge profiles of GCDs. In particular, the specific and volumetric capacitance (*C*, F g^−1^, and *C*_v_, F cm^−3^, respectively) of GO-StC electrodes were calculated from GCDs as follows:

3 $$C = \frac{{4\,I\,\Delta t}}{{m\,\Delta E}},$$

4 $$C_{{{{{{{\mathrm{v}}}}}}}} = \rho C,$$

where *I* is the current (A), Δ*t* is the discharge time (s), *m* is the total mass of both electrodes (g), Δ*E* is the potential window (V), and *ρ* is the density of the coating (g cm^−3^). The ESR (Ω) was estimated from the initial potential drop (*δE*) as follows:

5 $$ESR = \frac{{\delta E}}{{2\,{{{{{{{\mathrm{I}}}}}}}}}}$$

The gravimetric energy density (*U*, Wh kg^−1^) and the gravimetric power density (*P*, W kg^−1^) were obtained from the following equations:

6 $$U = \frac{{C\,E^2}}{{2 \times 3.6}},$$

7 $$P = \frac{U}{{\Delta t}},$$

EIS data were fitted by an equivalent circuit model approach using the software “ZView^®^” from Scibner Associates. An evaluation on the variation of the complex capacitance (*C*(*ω*)) with the frequency was also performed as follows^75^:

8 $$C\left( \omega \right) = C^\prime \left( \omega \right) - jC^{\prime\prime} \left( \omega \right),$$

9 $$C^{\prime} \left( \omega \right) = \frac{{ - Z^{\prime\prime} \left( \omega \right)}}{{\omega \left| {Z\left( \omega \right)} \right|^2}};C^{\prime\prime} \left( \omega \right) = \frac{{Z^{\prime} \left( \omega \right)}}{{\omega \left| {Z\left( \omega \right)} \right|^2}},$$

where *C*′(*ω*) and *C*″(*ω*) are the real and imaginary part of *C*(*ω*), respectively, and *ω* is the angular frequency. *Z*′(*ω*), *Z*″(*ω*), and |*Z*(*ω*)| are the real and imaginary part and the magnitude of the impedance.

## Supplementary information

Supplementary Information

## Data Availability

The data that support the findings of this study are available from the corresponding author upon reasonable request.
