# Supplementary material for: Graphene oxide and starch gel as a hybrid binder for environmentally friendly high-performance supercapacitors
Source: Commun Chem. 2021 Dec 6;4:169. doi: 10.1038/s42004-021-00604-0 (PMC9814468; doi:10.1038/s42004-021-00604-0)
Supplement: Supplementary file 1 — Supplementary Information [file 42004_2021_604_MOESM1_ESM.pdf]

Supplementary Information

Graphene Oxide and Starch Gel as a Hybrid Binder for Environmentally Friendly High-Performance Supercapacitors

Mario Rapisarda[1], Frank Marken[2], Michele Meo[1,*]

[1]Department of Mechanical Engineering, University of Bath, Bath, BA27AY, UK
[2]Department of Chemistry, University of Bath, Bath, BA27AY, UK

Supplementary Tables

Supplementary Table 1. Slurry composition of different GO-StC and reference electrodes.

Slurry name	AC (wt%)	CB (wt%)	St (wt%)	GO (wt%)
StC	85	5	10.00	0
GO-StC-I	85	5	7.50	2.50
GO-StC-II	85	5	6.67	3.33
GO-StC-III	85	5	5.00	5.00
GO-StC-IV	85	5	3.33	6.67
GO-StC-V	85	5	2.50	7.50
GO-C	85	5	0	10

Supplementary Table 2. Main peaks positions and calculated structural parameters from XRD patterns of GO-StC, StC, AC, GO, GO-St-gel, GO-St, and St (Fig. 2a of the manuscript). *d*, *B* and L_a are interplanar spacing in crystal lattice, line broadening at half-maximum intensity of the peak and crystallite lateral size, respectively.

Sample	(001) 2θ (°)	(002) 2θ (°)	(100) 2θ (°)	(10) 2θ (°)	$d_{(001) \text{ or } (002)}$ (Å)	$B_{(100) \text{ or } (10)}$ (°)	L_a (Å)
GO-StC	\	21.89	\	43.73	4.03	4.66	37.6
StC	\	22.24	\	43.49	3.99	4.07	43.0
AC	\	22.13	\	43.68	4.01	3.93	44.6
GO	10.85	\	42.57	\	8.15	1.36	128.0
GO-St-gel	\	\	42.62	\	\	2.01	86.9
GO-St	11.07	\	42.50	\	7.99	1.59	109.6

Supplementary Table 3. Main features and relative assignment from FT-IR spectra of GO, GO-St-gel, GO-St, and St (Fig. 2b of the manuscript).

**Code**	**Wavenumber (cm^{-1})**				**Assignment**[1, 2, 3]
	**GO**	**GO-St-gel**	**GO-St**	**St**	
1'	3586	-	3586	\	
1''	3216	-	3233	\	OH stretching
1	\	3286	-	3310	
2		\		2931	CH_2 bending
3		\		2894	
4	1720	1726	1722	\	C=O stretching (Carbonyl)
5		\		1642	OH bending and stretching of absorbed H_2O
6	1615	1627	1615	\	
7	1373	1356	1360	\	OH bending
8		\		1241	C-OH bending
9	1219	1230	1225	\	C-OH stretching
10		\		1207	CH_2 and C-OH bending
11	1165	-		\	C-OH stretching
12	\	1148	1150	1150	C-O/C-C/OH stretching and bending of glycosidic bridge
13		\		1078	
14		\		1042	C-OH bending
15	1038	-	1038	\	C-O stretching (Alkoxy)
16	\	1014	1015	1016	C-OH bending
17	\	998	993	995	C-O-C (Skeletal starch)
18	975	-		\	C-O-C stretching (Epoxy)

Supplementary Table 4. Interbands deconvolution of Raman spectra and resulting $I_D/(I_D+I_G)$ ratio of GO-StC, StC, AC, GO, GO-St-gel, and GO-St. Deconvolution result for GO is shown in Fig. 2d of the manuscript, GO-St-gel and GO-St show similar fittings. Deconvolution result for AC is shown in Fig. 2e of the manuscript, GO-StC and StC show similar fittings. x_c, w and I are peak position, full width at half height and peak height, respectively.

**Band [Fitting model]**	**Parameter**	**Sample**					
		GO-StC	**StC**	***AC***	***GO***	***GO-St***	***GO-St-gel***
*D** *[Gaussian]*	x_c (cm^{-1})				1231.5	1230.5	1243.6
	w (cm^{-1})		\		88.7	81.9	61.8
	I (counts)				874.4	943.0	1022.6
I *[Gaussian]*	x_c (cm^{-1})	1360.2	1350.1	1334.9			
	w (cm^{-1})	234.2	251.2	324.3		\	
	I (counts)	991.7	781.7	916.1			
D *[Ps-Voigt]*	x_c (cm^{-1})	1345.6	1345.5	1343.9	1353.4	1354.8	1351.0
	w (cm^{-1})	74.8	79.1	82.1	114.1	116.0	104.8
	I (counts)	2817.5	2560.4	3252.3	19953.7	23368.8	21706.5
D'' *[Gaussian]*	x_c (cm^{-1})	1557.6	1553.0	1554.2	1508.5	1510.0	1508.8
	w (cm^{-1})	105.2	108.3	109.3	149.1	145.6	148.4
	I (counts)	639.2	497.8	556.0	4381.8	5313.6	4051.0
G *[Ps-Voigt]*	x_c (cm^{-1})	1607.8	1606.8	1606.1	1587.9	1587.6	1591.0
	w (cm^{-1})	63.3	61.9	58.9	65.8	66.4	62.4
	I (counts)	2629.8	2323.0	2835.5	14119.8	16627.3	13686.1
D' *[Gaussian]*	x_c (cm^{-1})				1617.1	1617.6	1618.4
	w (cm^{-1})		\		38.8	39.0	38.1
	I (counts)				11505.5	13023.1	12741.6
**$I_D/(I_D+I_G)$ ratio (%)**		51.72	52.43	53.42	58.56	58.43	61.33

Supplementary Table 5. Starting, ending and peak temperatures of GO reduction and Starch degradation for GO-StC electrode material and GO-St-gel binder compared with reference materials (from TGA and dTGA analysis presented in Figure 3a-b of the manuscript).

**Parameter**	**Sample**						
	**GO-StC**	**StC**	**AC**	**GO**	**GO-St-gel**	**GO-St**	**St**
Reduction start (°C)	132			150	122	121	
Reduction end (°C)	-	\		300	-	-	\
Reduction peak (°C)	216			184	161	180	
Maximum red. rate (% / °C)	3.5			18	40	15	
Degradation start (°C)	~250–300	240			250	286	255
Degradation end (°C)	418	385	\		423	421	329
Degradation peak (°C)	No	320			313	347	293
Maximum deg. rate (%/°C)	~1.2	2.8			6.7	3.9	125.2

Supplementary Table 6 Main peaks positions and calculated structural parameters from XRD patterns of GO-StC coatings thermally treated at varying temperatures (Supplementary Fig. 1a). *d*, *B* and L_a are interplanar spacing in crystal lattice, line broadening at half-maximum intensity of the peak and crystallite lateral size, respectively.

**Treatment temperature (°C)**	**(002) 2θ (°)**	**(10) 2θ (°)**	$d_{(002)}$ **(Å)**	$B_{(10)}$ **(°)**	L_a **(Å)**
80	21.89	43.73	4.06	4.66	37.6
150	22.17	43.86	4.01	3.88	45.1
250	21.72	43.52	4.09	4.63	37.8
350	21.84	43.72	4.07	4.52	38.7
450	21.67	43.88	4.10	5.27	33.2
550	22.24	43.66	3.99	3.81	45.9

Supplementary Table 7. Interbands deconvolution of Raman spectra and resulting $I_D/(I_D+I_G)$ ratio of GO-StC coatings thermally treated at varying temperatures (Supplementary Fig. 1c). x_c, w and I are peak position, full width at half height and peak height, respectively.

Band [Fitting model]	Parameter	Treatment temperature (°C)					
		80	***150***	***250***	***350***	***450***	***550***
I [Gaussian]	x_c (cm^{-1})	1360.2	1343.6	1350.4	1365.0	1343.6	1342.9
	w (cm^{-1})	234.2	224.3	222.6	264.2	237.1	250.6
	I (counts)	991.7	967.8	630.9	1185.4	947.1	1052.3
D [Ps-Voigt]	x_c (cm^{-1})	1345.6	1345.2	1344.7	1346.3	1344.7	1344.3
	w (cm^{-1})	74.8	78.7	74.4	88.1	76.1	80.7
	I (counts)	2817.5	3156.9	2273.8	2216.0	3023.5	2980.2
D'' [Gaussian]	x_c (cm^{-1})	1557.6	1539.2	1547.1	1552.4	1551.5	1544.7
	w (cm^{-1})	105.2	100.4	112.7	114.0	111.6	107.3
	I (counts)	639.2	783.1	440.5	949.1	610.5	597.6
G [Ps-Voigt]	x_c (cm^{-1})	1607.8	1606.5	1606.8	1606.8	1606.1	1606.4
	w (cm^{-1})	63.3	58.7	61.8	64.3	59.9	60.2
	I (counts)	2629.8	3164.4	2014.8	2778.5	2713.9	3150.1
**$I_D/(I_D+I_G)$ ratio (%)**		51.72%	49.94%	53.02%	44.37%	52.70%	48.61%

Supplementary Table 8. Electrodes coating specifications (mass loading, m_l; thickness, *h*; density, *ρ*) and resulting specific and volumetric capacitance (*C* and C_v, respectively; calculated at 0.2 A g^{-1}).

Electrode	m_l (mg cm^{-2})	*h* (µm)	*ρ* (g cm^{-3})	*C* (F g^{-1})	C_v (F cm^{-3})
StC@80	2.94	117.52	0.25	105.9	26.5
GO-StC@80	2.81	114.17	0.25	125.0	30.7
rGO-StC@350	2.80	114.56	0.22	173.8	38.2

Supplementary Table 9. Fitted values of the simplified equivalent circuit model (Supplementary Fig. 4b) and capacitor properties extrapolated from experimental data for StC@80, GO-StC@80, and rGO-StC@350 supercapacitor symmetric cells.

Parameter		Supercapacitor symmetric cell		
		**StC@80**	**GO-StC@80**	**rGO-StC@350**
Simplified model fitting values	R_s [Ω]	6.83	4.43	4.12
	$C_{int\text{-}T}$ [s^n $Ω^{-1}$]	0.02	0.01	0.01
	$C_{int\text{-}P}$	0.59	0.45	0.45
	R_{ct} [Ω]	0.29	0.78	0.70
	W_R [Ω]	4.45	8.24	3.25
	W_T [s]	1.62	3.01	0.33
	W_P	0.62	0.53	0.47
	$C_{dl\text{-}T}$ [s^n $Ω^{-1}$]	0.08	0.13	0.27
	$C_{dl\text{-}P}$	0.61	0.83	1.00
Capacitor properties	*C'* at 0.01 Hz [F g^{-1}]	84.6	93.9	118.5
	Phase angle at 0.01 Hz [°]	-72.9	-77.2	-77.1
	Mid frequency transition [Hz]	0.32	0.32	6.31
	Response frequency at –45° [Hz]	0.14	0.21	0.31
	Relaxation frequency [Hz]	0.05	0.13	0.32

Supplementary Table 10. Comparison of specific capacitance and capacitance retention for different supercapacitors obtained using alternative green binder processable in water and conventional binders of commercially available devices. Some non-biomaterial based alternatives have also been included as reference. Water is used as the only solvent unless differently specified between brackets.

**Binder**	**Electrolyte**	**Potential window**	**Specific capacitance**	**Capacitance retention**	***Ref.****
rGO-St-gel (350 °C)	PVA/H_3PO_4 gel (60 wt%)	0–1 V	174 F g^{-1} at 0.2 A g^{-1}	93.1% [97.1%] after 17,000 [5,000] cycles at 4 A g^{-1}	This work
GO-St-gel (80 °C)	PVA/H_3PO_4 gel (60 wt%)	0–1 V	125 F g^{-1} at 0.2 A g^{-1}	92.5% after 5,000 cycles at 4 A g^{-1}	This work
St only (80 °C)	PVA/H_3PO_4 gel (60 wt%)	0–1 V	106 F g^{-1} at 0.2 A g^{-1}	92% after 5,000 cycles at 4 A g^{-1}	This work
Potato Starch	1 M Et_4NBF_4 in PC	0–1 V	54 F g^{-1} at ~0.5 A g^{-1}	97.2% after 5,000 cycles at ~1 A g^{-1}	15
CMC	1 M Et_4NBF_4 in PC	0–5 V	44 F g^{-1} at ~0.7 A g^{-1}	97.4% after 5,000 cycles at ~1.5 A g^{-1}	15
Potato Starch/ Guar Gum	1 M $TEABF_4$ in PC	0–2.5 V	26 F g^{-1} at 0.2 A g^{-1}	\	18
Starch glue	1 M $TEABF_4$ in ACN	0–2.5 V	~30 F g^{-1} at 0.5 A g^{-1}	~90% after 2,000 cycles at 0.5 A g^{-1}	14
Tragacanth Gum	1 M $TEABF_4$ in PC	0–2.75 V	23 F g^{-1} at 1 A g^{-1}	97% after 15,000 cycles at ~2 A g^{-1}	69
PVAc/poly(isoprene) [Xylene]	1 M Na_2SO_4 in H_2O	0–1 V	41 F g^{-1} at ~0.1 A g^{-1}	\	68
PVA/PVAc (crosslinked with $Na_2B_4O_7$)	1 M Na_2SO_4 in H_2O	0–1 V	64 F g^{-1} at ~0.1 A g^{-1}	\	68
Egg white	1 M Na_2SO_4 in H_2O	0–1 V	89 F g^{-1} at ~0.1 A g^{-1}	\	68
PTFE [Ethanol]	1 M Na_2SO_4 in H_2O	0–1 V	106 F g^{-1} at ~0.1 A g^{-1}	\	68
SBR/PTFE	1 M $TEABF_4$ in ACN	0–2.7 V	108 F g^{-1} at 0.2 A g^{-1}	~80% after 20,000 cycles at ~1 A g^{-1}	65-70
PVP [Ethanol]	1 M $TEABF_4$ in PC	0–2.7 V	112 F g^{-1} at 0.1 A g^{-1}	~81% after 10,000 cycles at 1 A g^{-1}	66-72
PTFE [NMP]	1 M $TEABF_4$ in PC	0–2.7 V	107 F g^{-1} at 0.1 A g^{-1}	~81% after 10,000 cycles at 1 A g^{-1}	66-72
Ch/poly(EG-ran-PG) [1% acetic acid in H_2O]	1 M H_2SO_4 in H_2O	0–1 V	172 F g^{-1} at 0.5 A g^{-1}	~99% after 6,000 cycles at 3.5 A g^{-1}	67-71

* Main manuscript reference number.

Supplementary Figures

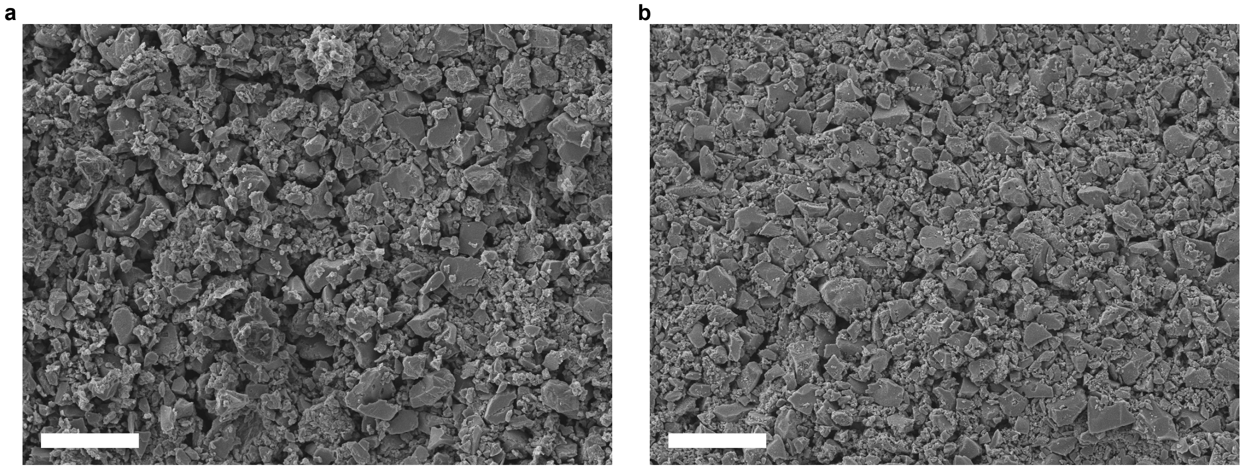

Supplementary Fig. 1 Electronic microscope imaging of GO-StC electrodes. a-b SEM images showing: surface morphology of **c** GO-StC and **d** StC coatings (scale bar 20 µm).

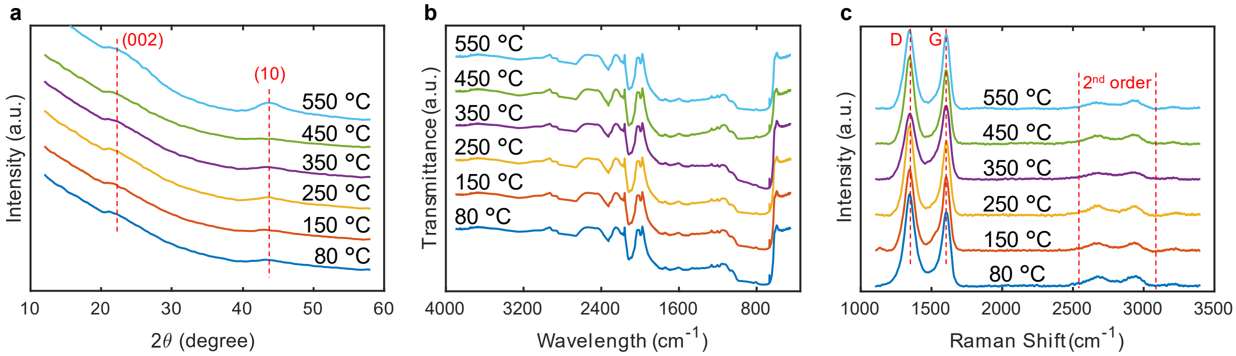

Supplementary Fig. 2 Physicochemical characterisation of GO-StC coatings at varying thermal treatment temperatures. a XRD patterns, **b** FT-IR and **c** RS spectra.

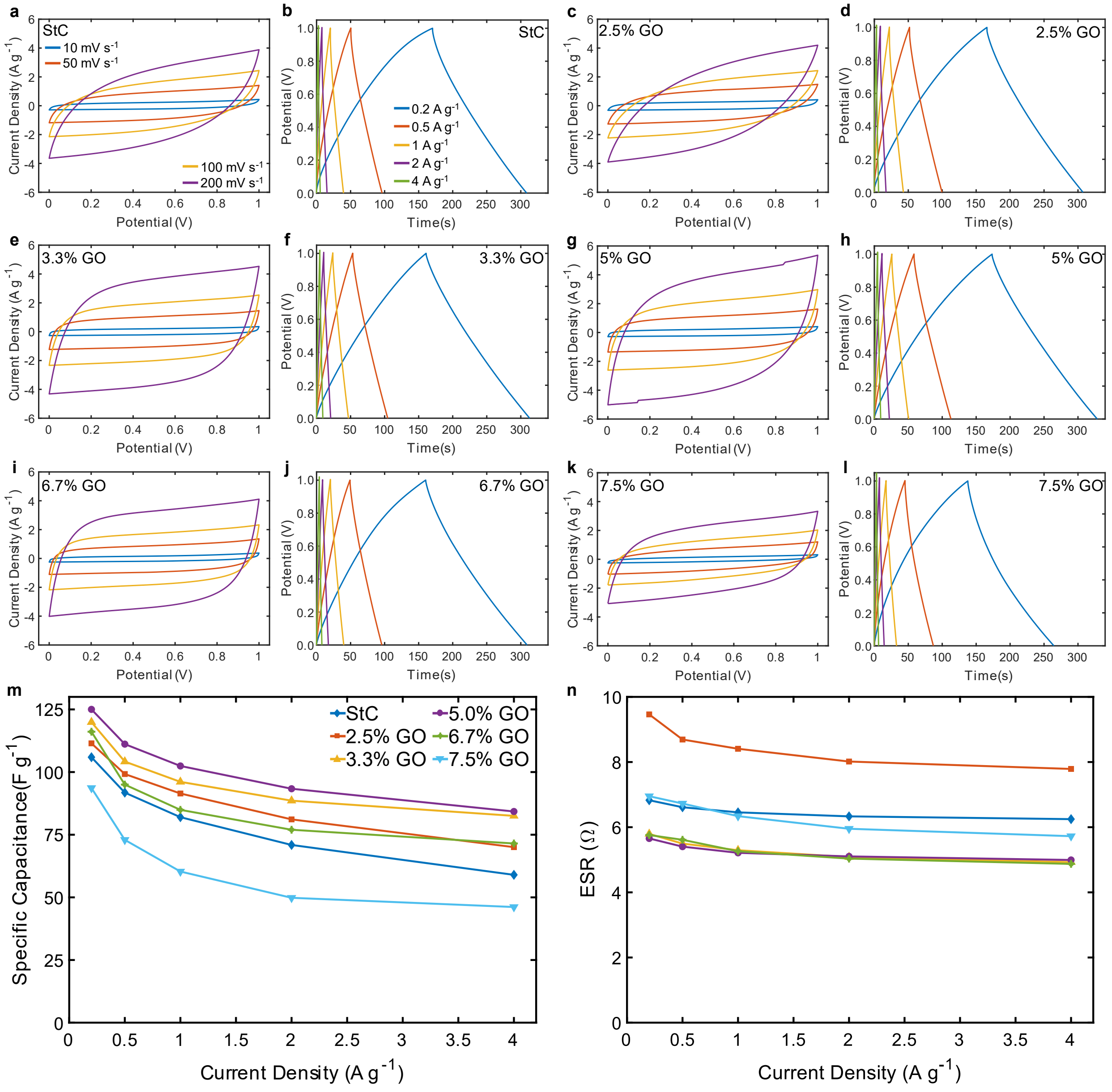

Supplementary Fig. 3 Electrochemical characterisation of GO-StC electrodes with GO amount varying from 0 (StC) to 7.5%. a, c, e, g, i, k Cyclic Voltammetry Scans (CVs) and **b, d, f, h, j, l** Galvanostatic Charge Discharge Cycles (GCDs). Same legend applies for all CVs and all GCDs. Variation of **m** specific capacitance and **n** Equivalent Series Resistance (ESR) with current density. Same legend applies for both panels.

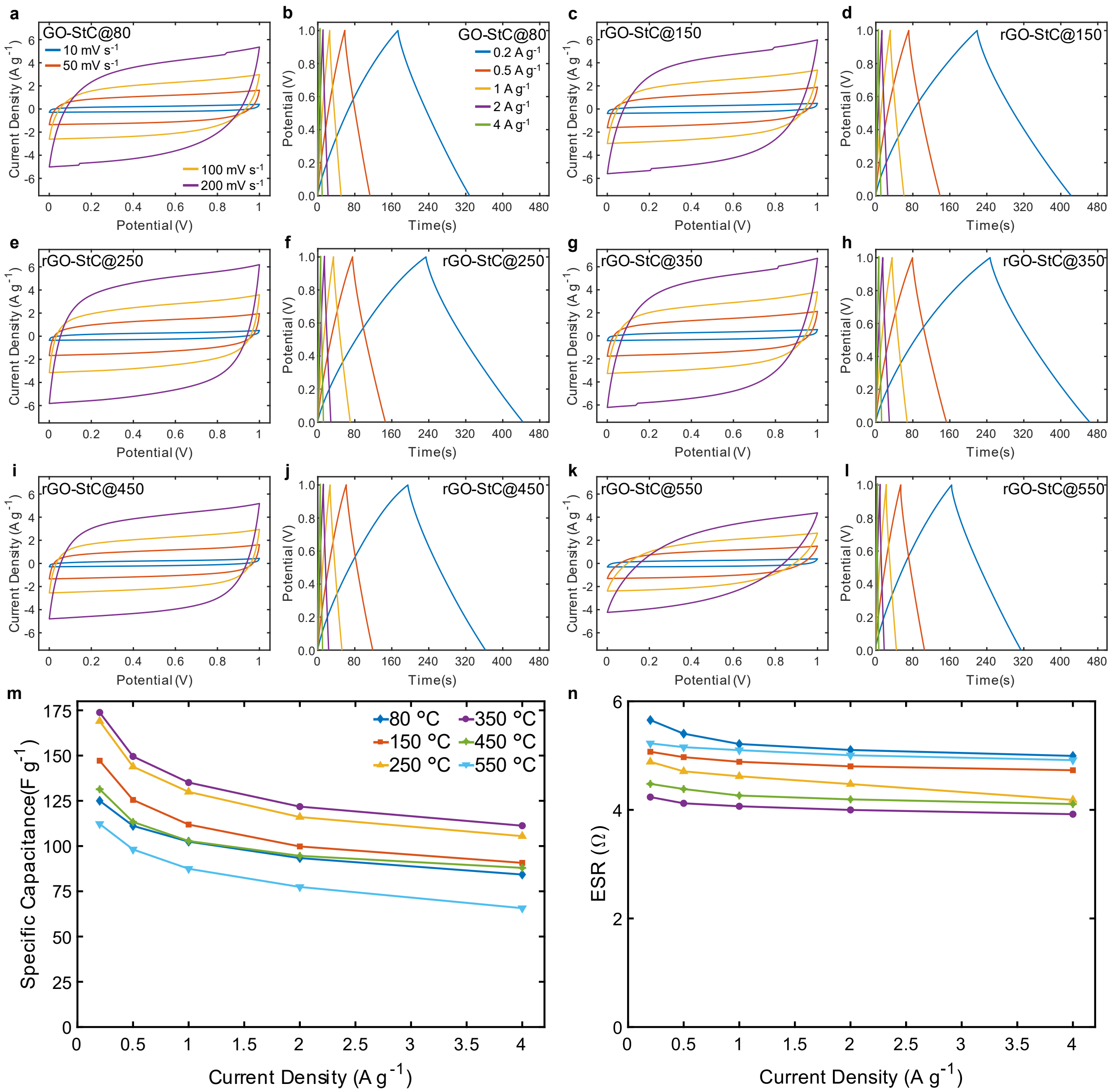

Supplementary Fig. 4 Electrochemical characterisation of GO-StC electrodes with thermal treatment temperatures varying from 80 to 550 °C. a, c, e, g, i, k Cyclic Voltammetry Scans (CVs) and **b, d, f, h, j, l** Galvanostatic Charge Discharge Cycles (GCDs). Same legend applies for all CVs and all GCDs. Variation of **m** specific capacitance and **n** Equivalent Series Resistance (ESR) with current density. Same legend applies for both panels.

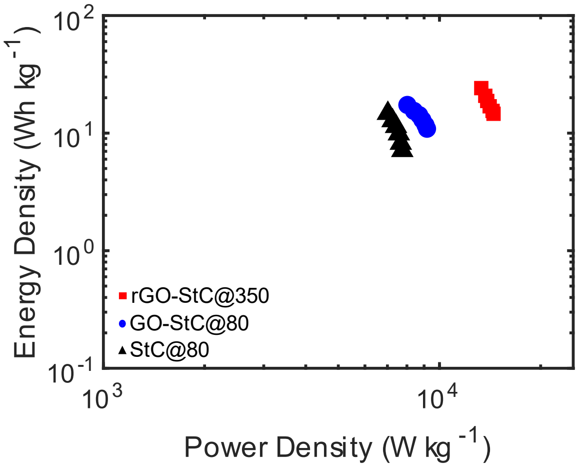

Supplementary Fig. 5 Ragone plot of assembled SCs.

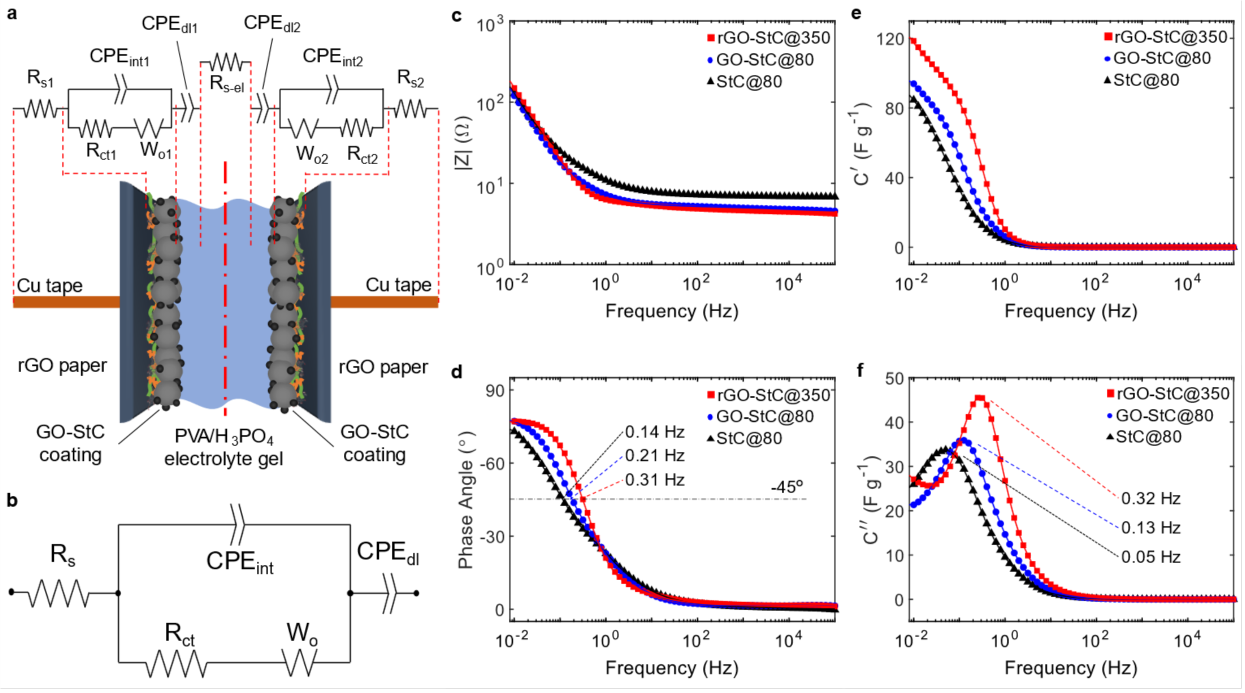

Supplementary Fig. 6 Impedance characterisation of supercapacitors. a Schematic of GO-StC symmetric cell with equivalent circuit elements assignment (R_{s1-2} are resistors accounting for leads, current collectors and interparticle series resistances; R_{s-el} accounts for the electrolyte series resistance; CPE_{int1-2} are constant phase elements accounting for interfacial non-ideal capacitances; R_{ct1-2} account for charge-transfer resistances at interfaces; W_{o1-2} are finite length-open Warburg elements accounting for the transmission-line behaviour of porous materials; CPE_{dl1-2} account for non-ideal double-layer capacitances[4]). **b** Simplified equivalent circuit for EIS fittings where $R_s = R_{s1} + R_{s2} + R_{s\text{-}el}$, $CPE_{dl} = (CPE_{dl1} + CPE_{dl2})/2$, $Z(R_{ct}\text{-}CPE_{int}\text{-}W_o) = Z(R_{ct1}\text{-}CPE_{int1}\text{-}W_{o1}) + Z(R_{ct2}\text{-}CPE_{int2}\text{-}W_{o2})$. **c-d** Bode plots of rGO-StC@350 with dried-only GO-StC and StC electrodes (GO-StC@80 and StC@80) included for comparison. Markers represents the experimental points, while solid lines the modelled behaviour with the simplified equivalent circuit. Capacitors response frequency at a phase angle of -45° are specified. Variation of **e** Real and **f** imaginary part of complex capacitance for StC@80, GO-StC@80, and rGO-StC@350. Capacitors relaxation frequency (local maxima of *C''*) are specified.

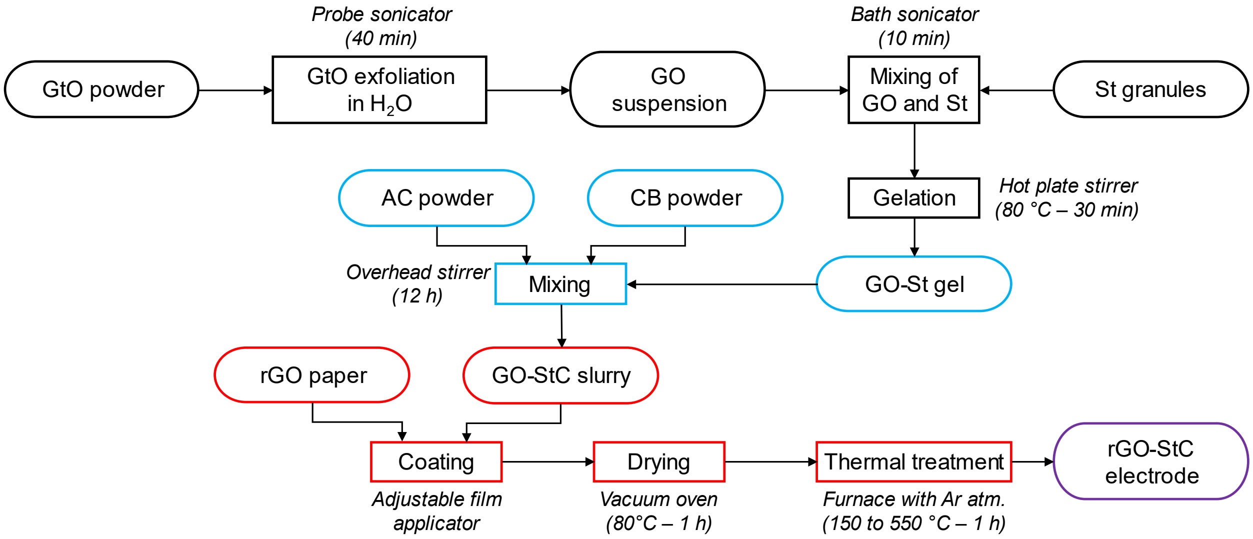

Supplementary Fig. 7 Flow diagram of rGO-StC electrodes fabrication process.

Supplementary Reference

1. Cael JJ, Koenig JL, Blackwell J. Infrared and Raman spectroscopy of carbohydrates. Part VI: Normal coordinate analysis of V-amylose. *Biopolymers* **14**, 1885-1903 (1975).

2. Titelman GI, Gelman V, Bron S, Khalfin RL, Cohen Y, Bianco-Peled H. Characteristics and microstructure of aqueous colloidal dispersions of graphite oxide. *Carbon* **43**, 641-649 (2005).

3. Zhang J, Yang H, Shen G, Cheng P, Zhang J, Guo S. Reduction of graphene oxide vial-ascorbic acid. *Chemical Communications* **46**, 1112-1114 (2010).

4. Conway BE. *Electrochemical Supercapacitors*, 1 edn. Springer (1999).
